# 213. Prophylactic oral vancomycin for *Clostridioides difficile* in allogeneic hematopoietic stem cell transplant recipients

**DOI:** 10.1093/ofid/ofac492.290

**Published:** 2022-12-15

**Authors:** Lauren Ogawa, Kaylor Wright, Alfred Luk

**Affiliations:** Tulane University, Los Angeles, California; Tulane University, Los Angeles, California; Tulane University, Los Angeles, California

## Abstract

**Background:**

Allogeneic hematopoietic stem cell transplant (alloHSCT) recipients are at substantial risk for *Clostridioides difficile* infection (CDI) due to prolonged hospitalization and alteration of the intestinal mucosa and gut microbiota from conditioning regimens, antimicrobials, and immunosuppressants. CDI in alloHSCT recipients has also been associated with higher rates of acute graft versus host disease (aGVHD). Review of the literature revealed that three institutions have published studies demonstrating oral vancomycin prophylaxis prevents CDI. However, more studies are needed to evaluate the impact of prophylaxis on CDI incidence, resistant infections, and other transplant-related outcomes.

**Methods:**

We conducted a retrospective review to compare the effectiveness of prophylactic oral vancomycin versus no prophylaxis in 40 alloHSCT recipients in the prevention of CDI at our institution. The prophylactic intervention was initiated June 2020, and 20 patients have undergone alloHSCT as of April 2022. Patients received twice daily oral vancomycin starting the day of admission for transplant until discharge. The prophylaxis group was compared to a control group of 20 consecutive alloHSCT recipients prior to initiation of the intervention. The primary outcome was the incidence of CDI. Secondary outcomes included incidence of vancomycin resistant enterococcus (VRE) infection and incidence of aGVHD.

**Results:**

During admission for transplant and at +100 days post-transplant, 5/20 (25%) of patients in the control group developed CDI compared to 0/20 (0%) in the prophylaxis group (p=0.0471). Prophylaxis was not associated with higher rates of VRE infection through day +100 post-transplant (2/20, 10% vs 0/20, 0%, p=0.487). Use of oral vancomycin was not associated with increased incidence of aGVHD, which occurred in 10/20 (50%) of patients in the control group versus (7/20) 35% in the prophylaxis group (p=0.523).

**Conclusion:**

Prophylaxis with oral vancomycin was associated with a significant reduction in CDI. Use of prophylaxis was not associated with an increased risk for VRE infection and there was no change in incidence of aGVHD. Our review supports the use of prophylaxis, and we will continue to monitor for resistant infections and evaluate the impact on survival.

**Disclosures:**

**Alfred Luk, MD**, Karius: Grant/Research Support.

